# Comprehensive time-course gene expression evaluation of high-risk beef cattle to establish immunological characteristics associated with undifferentiated bovine respiratory disease

**DOI:** 10.3389/fimmu.2024.1412766

**Published:** 2024-09-13

**Authors:** Matthew A. Scott, Robert Valeris-Chacin, Alexis C. Thompson, Amelia R. Woolums, Brandi B. Karisch

**Affiliations:** ^1^ Veterinary Education, Research, and Outreach Program, Texas A&M University, Canyon, TX, United States; ^2^ Texas A&M Veterinary Medical Diagnostic Laboratory, Canyon, TX, United States; ^3^ Department of Pathobiology and Population Medicine, College of Veterinary Medicine, Mississippi State University, Mississippi State, MS, United States; ^4^ Department of Animal and Dairy Sciences, Mississippi State University, Mississippi State, MS, United States

**Keywords:** cattle, bovine respiratory disease, inflammation, specialized pro-resolving mediators, major histocompatibility complex, immunoglobulin, interleukin, transcriptome

## Abstract

Bovine respiratory disease (BRD) remains the leading infectious disease in beef cattle production systems. Host gene expression upon facility arrival may indicate risk of BRD development and severity. However, a time-course approach would better define how BRD development influences immunological and inflammatory responses after disease occurrences. Here, we evaluated whole blood transcriptomes of high-risk beef cattle at three time points to elucidate BRD-associated host response. Sequenced jugular whole blood mRNA from 36 cattle (2015: *n* = 9; 2017: *n* = 27) across three time points (*n* = 100 samples; days [D]0, D28, and D63) were processed through ARS-UCD1.2 reference-guided assembly (HISAT2/Stringtie2). Samples were categorized into BRD-severity cohorts (Healthy, *n* = 14; Treated 1, *n* = 11; Treated 2+, *n* = 11) via frequency of antimicrobial clinical treatment. Assessment of gene expression patterns over time within each BRD cohort was modeled through an autoregressive hidden Markov model (EBSeq-HMM; posterior probability ≥ 0.5, FDR < 0.01). Mixed-effects negative binomial models (glmmSeq; FDR < 0.05) and edgeR (FDR < 0.10) identified differentially expressed genes between and across cohorts overtime. A total of 2,580, 2,216, and 2,381 genes were dynamically expressed across time in Healthy, Treated 1, and Treated 2+ cattle, respectively. Genes involved in the production of specialized resolving mediators (SPMs) decreased at D28 and then increased by D63 across all three cohorts. Accordingly, SPM production and alternative complement were differentially expressed between Healthy and Treated 2+ at D0, but not statistically different between the three groups by D63. Magnitude, but not directionality, of gene expression related to SPM production, alternative complement, and innate immune response signified Healthy and Treated 2+ cattle. Differences in gene expression at D63 across the three groups were related to oxygen binding and carrier activity, natural killer cell-mediated cytotoxicity, cathelicidin production, and neutrophil degranulation, possibly indicating prolonged airway pathology and inflammation weeks after clinical treatment for BRD. These findings indicate genomic mechanisms indicative of BRD development and severity over time.

## 1 Introduction

Bovine respiratory disease (BRD) remains the leading disease complex in North American beef cattle feeding systems (1, 2). In addition to being the leading cause of clinical intervention and mortality in feeder systems, clinical and subclinical BRD reduces feed efficiency, growth performance, and carcass quality, making it the costliest infectious disease process in post-weaned beef cattle systems (3–8). As a polymicrobial, multifactorial disease complex, BRD stems from the interplay of viral and bacterial pathogenic airway establishment, environmental stressors, and management factors (7–10). However, despite research and advancements in management tactics and diagnostics, BRD is commonly identified via undifferentiated clinical diagnosis and may be underreported antemortem (11–14). Moreover, concurrent prognostic and diagnostic testing fail to achieve early detection and targeted intervention, and the problems faced and/or priorities of individual feeding systems influence the utility and implementation of BRD mitigation tactics (11, 12, 15–17).

To provide potential novel methods and biomarkers to more accurately identify cattle that develop and/or succumb to BRD, our research group and others have focused on evaluating the host transcriptomes of cattle at the time of facility arrival with respect to naturally occurring BRD outcomes to identify potential indicators of concurrent subclinical or eventual development of BRD (18–21). Likewise, several research groups have evaluated cattle transcriptomes in case-control studies with regard to naturally occurring BRD in an effort to understand key molecular events and signaling pathways involved in respiratory disease development and clinical presentation (18, 22–24). Collectively, these studies have identified immune system-driven biological mechanisms, such as type-I interferon production, complement, and inflammatory mediation, in relative gene expression levels that appear to indicate BRD outcomes and potential severity. However, relatively few studies have assessed the impact that BRD acquisition has on the host immune response over time, particularly within the context of disease resolution (18).

Accordingly, we sought to analyze the transcriptomes of high-risk stocker cattle, enrolled over multiple years, to identify potential changes in immune function and signaling pathways associated with BRD development. Here, we hypothesized that BRD development (1) could be identifiable with host gene expression signaling upon arrival, defined by previously identified genes and enriched pathways (18–21), and (2) impacts the immune system in a prolonged and identifiable manner following disease resolution. The goal of this work is to provide key insights into the immunological influence and signaling influenced by BRD, which may impact health and performance outcomes when cattle are transitioned into feedlot or finisher production systems. Here, our findings provide a foundation for understanding immunological signaling influenced by respiratory disease development and resolution, which may be leveraged in future disease-mitigation research and approaches.

## 2 Materials and methods

### 2.1 Animal use approval and sample selection

Animal use and procedures were approved by the Mississippi State University Animal Care and Use Committee (IACUC Protocol No. 15-003 and No. 17-120) and carried out in accordance with relevant IACUC and agency guidelines and regulations. The information reported here is in accordance with Animal Research: Reporting of *In Vivo* Experiments (ARRIVE) guidelines (25).

The primary focus of this study was to determine gene expression patterns over time that were influenced by naturally occurring clinical BRD. The cattle used in this experiment were included in a multi-year (2015, 2017) clinical trial to determine the effect of vaccine and anthelminthic administration upon arrival on health and performance outcomes, compared in a 2 × 2 factorial design; complete study information, including treatment and pen allocation, feed and mineral source, and sampling schedule, is found elsewhere (26, 27). A total of 160 cattle from both years (*n* = 80, 2015; *n* = 80, 2017), confirmed to be free of bovine viral diarrhea virus persistent infection via ear notch antigen-capture ELISA, were housed and maintained in an identical fashion at the Leveck Animal Research Facility at Mississippi State University for 85 and 82 days for the 2015 and 2017 groups, respectively. For the 2015 study, jugular whole blood from cattle was collected into Tempus RNA blood tubes (Applied Biosystems, Waltham, MA, USA) at days 0, 14, 28, and 70. For the 2017 study, whole blood was collected in an identical fashion at days 0, 26 (referred to as 28 for simplicity), and 54. All blood samples were stored at − 80°C until analysis. Individual body weights were collected every 2 weeks in each study. All cattle were assessed for visual signs of clinical BRD and/or other disease processes by trained staff, where signs for BRD were assigned a severity score of 0–4 (0 = normal, 1 = mild, 2 = moderate, 3 = severe, 4 = moribund), closely resembling the scoring system previously described (28, 29). Antimicrobial treatment for BRD was instituted as described by Griffin and colleagues (26). Cattle deemed unlikely to recover were euthanized by project veterinarians via intravenous administration of pentobarbital, followed by a gross necropsy that was performed by trained staff. Following the conclusion of both studies, cattle were grouped based on the frequency of clinical treatment for BRD: never diagnosed nor treated (Healthy), treated one time throughout the course of their study (Treated 1), and treated twice or more and/or succumbed to clinical BRD (Treated 2+). Notably, only one individual included in this study (ID33_2015) succumbed to naturally occurring BRD and was found acutely dead on day 51 of the 2015 study (19).

Our primary objective for sample selection was to enroll random individuals while stratifying for relatively equal numbers of samples across treatment frequency groups, time points, and years. For this study, we utilized later time points from cattle whose at-arrival (D0) samples had been previously sequenced for the identification of candidate genes and mechanisms associated with BRD outcomes (19, 20) and randomly included nine additional cattle not previously transcriptomically evaluated at D0 (ID_20_2015, ID_40_2015, ID_60_2015, ID_68_2015, ID_71_2015, ID_114_2017, ID_147_2015, ID_166_2017, and ID_192_2017) to increase statistical power for differential gene expression detection. An *a priori* power analysis was performed with RnaSeqSampleSize (30) to calculate assumed study power with our combined RNA-Seq data set, utilizing the following parameters based on outcomes from our previous work (19, 20): minimum number of biological replicates per treatment frequency group (Healthy, Treated 1, and Treated 2+) of 11, minimum average read counts among prognostic genes of 200, maximum estimated gene-wise dispersion of 0.4, the ratio of the geometric mean of normalization factors of 1.5, total number of filtered genes to test across of 16,000, the top 200 genes being prognostic, desired minimum log2 fold-change of prognostic genes set to 2.0, and an established threshold of significance (FDR) of 0.10; statistical probability utilizing Exact test procedures resulted in a power of 0.81. As jugular blood sampling across these two populations was nonsequential when compared to each other, we elected to match the D0 and D28 time point samples from each year and include D70 samples from 2015 (26) and D54 samples from 2017 (27); the D70 and D54 samples were the furthest time points in which Tempus RNA blood tubes were collected from the 2015 and 2017 populations, respectively. For simplicity, these final sampling time points are referred to as D63, the average day of the final time point per sample collection. As peak incidence of BRD is often assumed to occur within the first 2–4 weeks of arrival in stocker cattle systems, D28 and D63 time points were selected to evaluate host gene expression changes at the point in which BRD likelihood would begin to decrease and at the relative end of the stocker production cycle when cattle are typically marketed for feedlot production system entry. We selected 73 samples from 36 individuals for RNA extraction and sequencing. The raw sequencing data from these samples and the corresponding raw sequencing D0 data from our previous experiments (19, 20), for a total of 100 samples from 36 individuals (*n* = 14, Healthy; *n* = 11, Treated 1; *n* = 11, Treated 2+) across three time points (D0, D28, D63), were analyzed. Complete metadata for the cattle enrolled in this study are found in **Supplementary Table S1**.

### 2.2 Sample processing and bioinformatic workflow of raw reads

To maintain similarity between RNA-Seq projects, the newly included 73 samples were processed in an equivalent manner to our previous work (19, 20). Total RNA isolation was performed with Tempus Spin RNA Isolation Kit (Applied Biosystems), per the manufacturer’s instructions. Following extraction, samples were evaluated for concentrations and RNA integrity via Qubit 4 fluorometry with RNA Broad Range quantification assay kits (ThermoFisher, Waltham, MA, USA) and TapeStation 4200 electrophoresis with RNA ScreenTapes and analysis reagents (Agilent, Santa Clara, CA, USA), respectively. One sample (ID68_D63) required RNA concentration via a Savant SpeedVac DNA 130 Integrated Vacuum Concentrator System (ThermoFisher, Waltham, MA, USA). All RNA samples were of acceptable quality (RIN: 6.0–9.7; mean = 9.1, SD = 0.6) and concentrations (ng/μL: 29.1–482.0; mean = 188.4, SD = 66.9) to proceed for sequencing library preparation. Library preparation and sequencing were performed by the Texas A&M University Institute for Genome Sciences and Society (TIGSS; College Station, TX, USA). Library preparation for mRNA was performed with the Stranded mRNA Prep Kit (Illumina, San Diego, CA, USA), following the manufacturer’s instructions. Paired-end sequencing for 150 base-pair read fragments was performed on an Illumina NovaSeq 6000 analyzer (v1.7+; S4 reagent kit v1.5) in one flow cell lane.

The 73 newly sequenced raw reads were processed bioinformatically together with the D0 samples from 2015 (19) and 2017 (20), in an effort to reduce technical bias across projects; the 2015 and 2017 samples were previously submitted and are found at the National Center for Biotechnology Information Gene Expression Omnibus (NCBI-GEO), under the accession numbers GSE136176 and GSE161396, respectively. Raw sequencing data for the 73 newly processed samples in this study are available at the NCBI-GEO under the accession number GSE194167. Raw reads were quality assessed with FastQC v0.11.9 (https://www.bioinformatics.babraham.ac.uk/projects/fastqc/) and MultiQC v1.12 (31). Trimmomatic v0.39 (32) was used to perform read pair trimming, adaptor removal, and minimal quality retainment with the following parameters: leading/trailing bases were removed if below a base Phred quality score of 3, removal of reads with 4 base-pair sliding window having a Phred quality score of less than 20, and removal of read fragments with a length below 32 bases. Retained reads were mapped and indexed to the bovine reference genome assembly ARS-UCD1.2 with HISAT2 v2.2.1 (33). Sequence Alignment/Map (SAM) files were converted to Binary Alignment Map (BAM) files prior to transcript assembly via Samtools v1.14 (34). Transcript assembly and gene-level expression estimation for downstream analyses were performed with StringTie v2.2.0 (35, 36), as described by Pertea and colleagues (37).

### 2.3 Differential gene expression analysis

Gene-level count matrices were processed and analyzed with R v4.1.2. To control batch effects, the package ComBat-seq [sva] v3.42.0 (38) was used to account for the sequencing platform across all samples. Briefly, the variable “Platform” (**Supplementary Table S1**) was used in ComBat-seq as the batch effect for each sample, indicated as the numeral “1” for all newly sequenced samples, “2” for GSE136176 samples, and “3” for GSE161396 samples, and “Severity” (**Supplementary Table S1**) was used as the biological condition of interest, with all other parameters set to default. Samples were further processed and filtered to reduce data sparsity by procedures described by Chen and colleagues (39), where any gene with a minimum total count above 100 and a count-per-million (CPM) of 0.5 in at least twelve samples was retained for further analysis.

Raw, filtered gene-level counts were normalized across samples with the trimmed mean of *M*-values (TMM) method (40) and had tagwise dispersion estimates calculated for input into glmmSeq v0.2.2 (https://github.com/myles-lewis/glmmSeq) for negative binomial mixed-effect time-course evaluation. Model adaptation allowed for the assessment of differentially expressed genes (DEGs) across time points, BRD severity groups (by frequency of antimicrobial therapy), and the interactions between time points and severity groups, where *p*-values were adjusted to control the false discovery rate (FDR) with the Benjamini–Hochberg method; genes were considered significantly expressed with an FDR < 0.05. The following negative binomial mixed-effect model was fitted to account for the three time points (“Day”) and treatment frequencies (“Severity”) as fixed effects, and individual (“actual_ID”), population (“Year”), and at-arrival vaccination (“Vax”) as random intercepts:

Model = gene count ~*Day* + *Severity* + *Day*: *Severity* + (1|*actual_ID*) + (1|*Year*) + (1|*Vax*)

Pairwise comparisons for DEGs between each severity group at each time point were performed with raw, filtered gene-level counts with edgeR v3.36.0 (41, 42). Within edgeR, genes were fitted under a generalized linear model (GLM) framework and analyzed via quasi-likelihood *F*-tests (QLF), following common dispersion estimation and blocking for a year and individual ID. Pairwise gene comparisons were considered significant with an FDR < 0.10.

### 2.4 Dynamic gene expression trend analyses

Gene expression dynamics over time were evaluated for each disease severity group (“Healthy”, “Treated 1”, “Treated 2”) independently. First, library size factors were calculated for raw filtered counts with the Upper-Quartile normalization method (43). Following normalization procedures, gene expressional paths over time were identified for each severity group through time-series differential analysis via EBSeq-HMM v1.28.0 (44). Briefly, EBSeq-HMM utilizes an autoregressive hidden Markov model to categorize expressional dependence over ordered conditions (time points D0, D28, and D63 in the case of this study) and identifies dynamic paths where gene expression continuously changes. For this study, we specifically retained genes possessing continuous changes (i.e., up- or downregulation) between each time point, with the four pathways described as “Up-Down” (for upregulation from D0 to D28, then downregulation from D28 to D63), “Up-Up” (for upregulation from D0 to D28, and continued upregulation from D28 to D63), “Down-Down” (downregulation from D0 to D28, and continued downregulation from D28 to D63), or “Down-Up” (downregulation from D0 to D28, then upregulation from D28 to D63). EBSeq-HMM was performed with 100 iterations of the Baum–Welsh algorithm (“UpdateRd=100”), an expected fold-change of 2.4 (“FCV=2.4”), two-chain mixture proportion updating set to FALSE (“UpdatePI=FALSE”), and all other parameters set to default. These parameters were set after 500 testing runs to maximize the log-likelihood estimates and eventual model fitting, per the developer’s instruction (https://www.bioconductor.org/packages/release/bioc/vignettes/EBSeqHMM/inst/doc/EBSeqHMM_vignette.pdf). The resulting maximized log-likelihood estimates and treatment plots for all tested parameters are found in **Supplementary Table S2**. Dynamically expressed genes were retained, having a posterior probability ≥ 0.50 and FDR < 0.01.

### 2.5 Gene-level functional enrichment analyses

Biological pathway and Gene Ontology (GO) term analyses were performed in the KOBAS-intelligence v3.0 API framework (accessed 20 May 2023) from DEGs found by (1) glmmSeq Severity and edgeR QLF testing at each time point and (2) glmmSeq Day: Severity interactions and the compiled list of DEGs from edgeR QLF testing (45). Within KOBAS-i, input genes were analyzed via the overrepresentation analysis method using the hypergeometric distribution, Fisher’s exact testing, and the *Bos taurus* genome as the background species reference. Specifically, functional enrichments within KOBAS-i utilized the GO knowledgebase, Kyoto Encyclopedia of Genes and Genomes, and Reactome databases (46–48). The Benjamini–Hochberg procedure was used to control the FDR, and any functional enrichment having an FDR < 0.05 was considered significant. An upset plot was generated to visualize the overlap of DEGs and functional enrichment terms identified between severity groups across each time point via Intervene (49, 50).

In an effort to avoid overdetection of terms and pathways enriched by DEGs by the same functional enrichment toolset (51–53), we elected to perform analyses with the directionally expressed genes identified by EBSeq-HMM with g:Profiler ve110_eg57_p18_4b54a898 (accessed 2 January 2024) (54). Here, genes identified in each directional pathway in each BRD severity group were imputed as the conserved gene name identified by the *Bos taurus* genome reference database. In g:Profiler, genes were input as an ordered query, ranked in descending order by maximum posterior probability (**Supplementary Table S4**), and the parameters included only annotated genes, the GO molecular function, cellular component, and biological process, KEGG, Reactome, and WikiPathways as the data source background, and applying the g:SCS multiple test correction technique with an adjusted *p*-value cutoff of 0.05 (54–57). All other parameters were set to default.

## 3 Results

### 3.1 Determination of significant gene expression patterns

**Figure 1:**
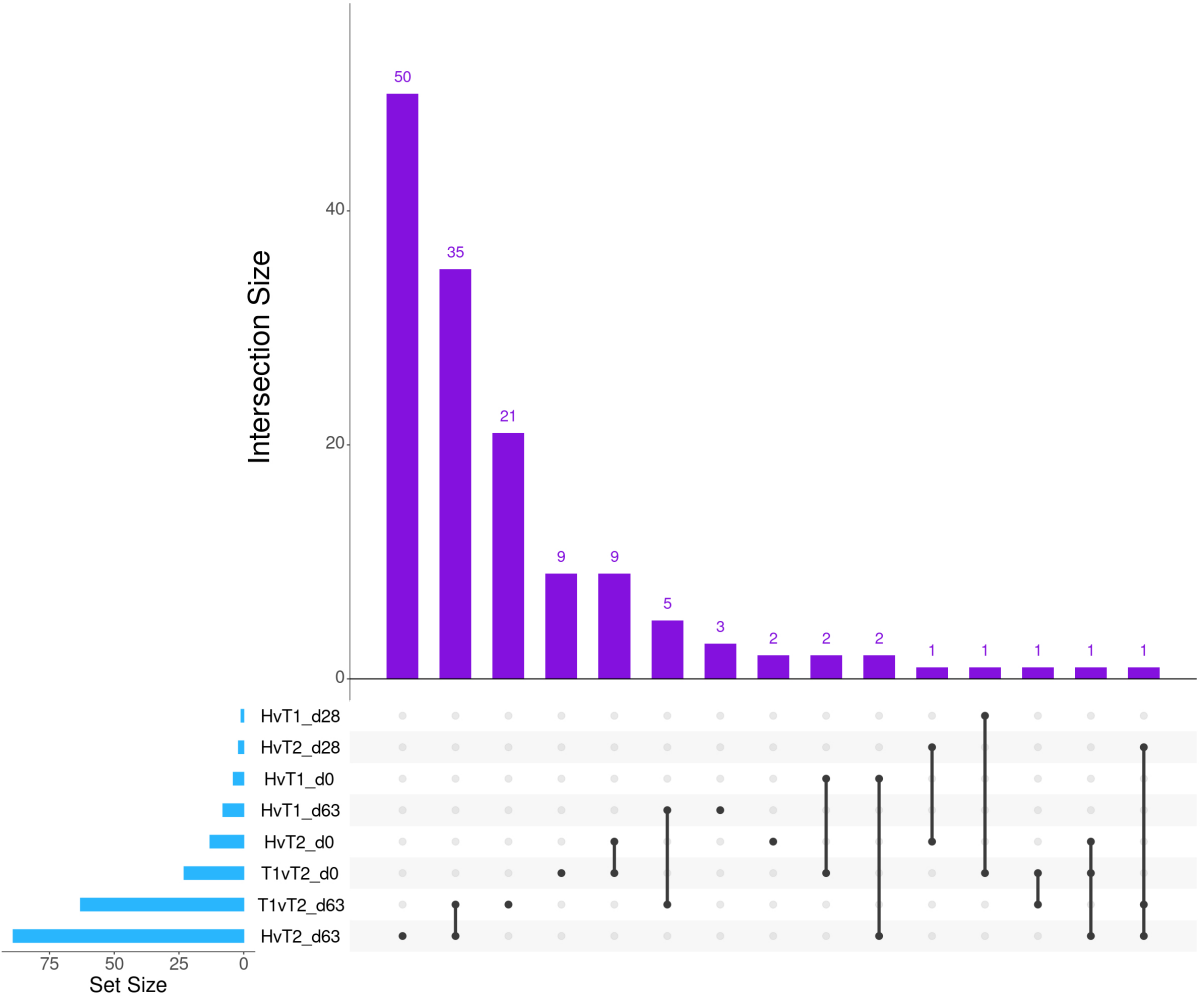
Matrix intersections of the number of differentially expressed genes (set size) identified between severity groups by edgeR and glmmSeq analyses. Each column represents the number of genes (intersection size) corresponding to one or more intersecting sets of analysis (row). Healthy cattle are indicated by “H”, Treated 1 cattle by “T1”, and Treated 2+ cattle by “T2”. Each day of sampling (0, 28, 63) is indicated by “d0”, “d28”, or “d63”.

Differential gene expression analysis with both edgeR and glmmSeq resulted in 3,257 uniquely identified DEGs between treatment groups over time (**Supplementary Table S3**). Following the overlapping of results between the two analysis platforms, a total of 203 and 286 DEGs were identified when evaluating the differences between each treatment group at each time point and the interaction of Severity and Time, respectively (**Table 1**). The overlaps of DEGs (gene names) identified within each time point are found in **Figure 1**. Here, the most unique DEGs were found between Healthy vs. Treated 2+ cattle at D63 (*n* = 50), Treated 1 vs. Treated 2+ cattle at D63 (*n* = 21), and Treated 1 vs. Treated 2+ cattle at Day 0 (*n* = 9). The greatest number of overlapping DEGs were observed between Healthy vs. Treated 2+ and Treated 1 vs. Treated 2+ at Day 63 (*n* = 35), Treated 1 vs. Treated 2+ and Healthy vs. Treated 2+ at Day 0 (*n* = 9), and Treated 1 vs. Treated 2+ and Healthy vs. Treated 1 at Day 63 (*n* = 5); no overlap was observed at Day 28.

**Table 1:** Total number of differentially expressed genes identified for each comparative analysis via edgeR and glmmSeq.

Comparison	DEGs
Healthy vs. Treated 1 (Day 0; glmmSeq + edgeR)	4
Healthy vs. Treated 2+ (Day 0; glmmSeq + edgeR)	13
Treated 1 vs. Treated 2+ (Day 0; glmmSeq + edgeR)	23
Healthy vs. Treated 1 (Day 28; glmmSeq + edgeR)	1
Healthy vs. Treated 2+ (Day 28; glmmSeq + edgeR)	2
Treated 1 vs. Treated 2+ (Day 28; glmmSeq + edgeR)	0
Healthy vs. Treated 1 (Day 63; glmmSeq + edgeR)	8
Healthy vs. Treated 2+ (Day 63; glmmSeq + edgeR)	89
Treated 1 vs. Treated 2+ (Day 63; glmmSeq + edgeR)	63
Severity : Time interaction (glmmSeq + edgeR)	286

**Figure 2:**
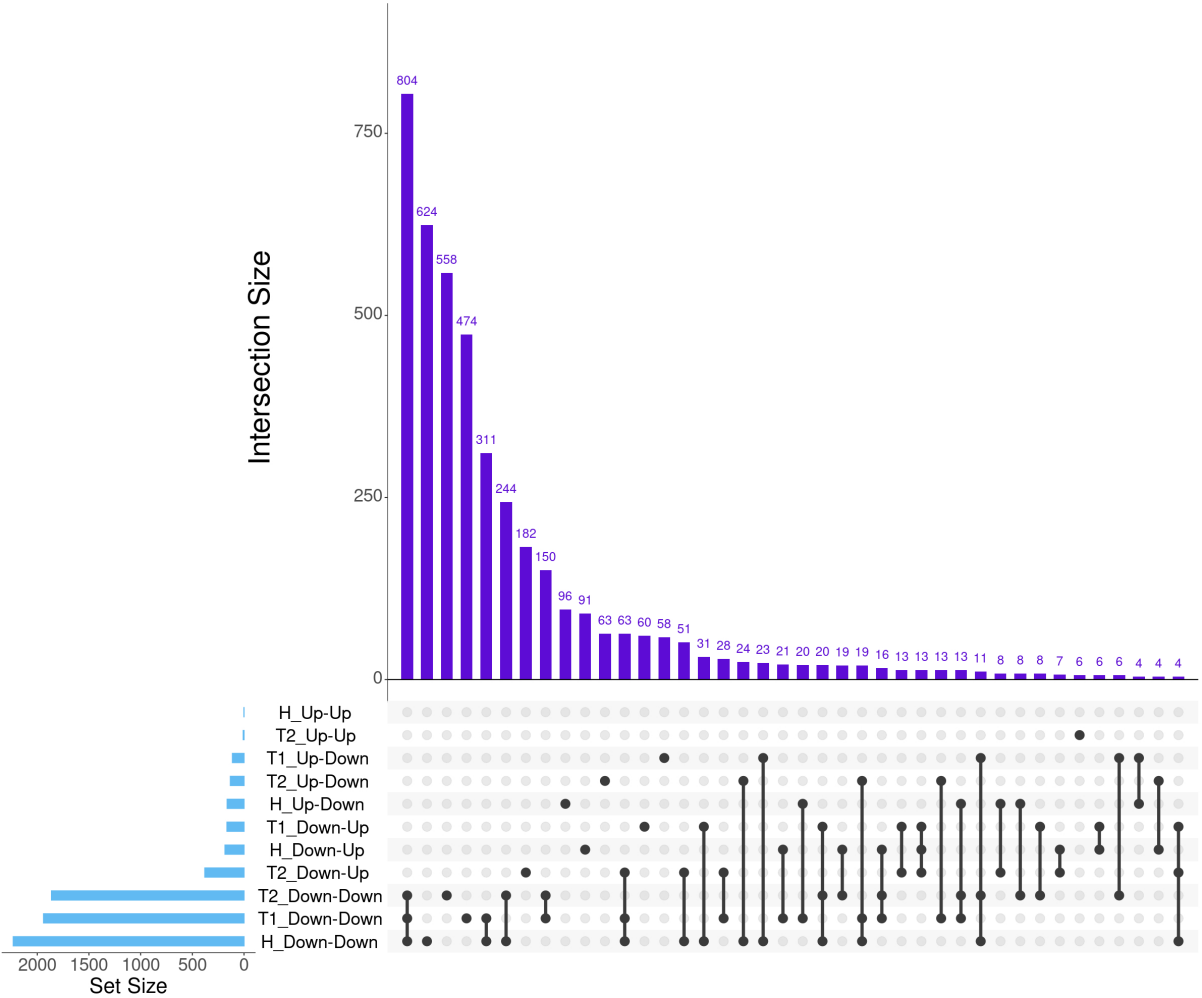
The top 40 matrix intersections of dynamically expressed genes (set size) identified between severity groups by EBSeq-HMM analyses. Each column represents the number of genes (intersection size) corresponding to one or more intersecting sets of analysis (row). Healthy cattle are indicated by “H”, Treated 1 cattle by “T1”, and Treated 2+ cattle by “T2”. Gene expression directionality is indicated by Up-Up, Up-Down, Down-Up, and Down-Down.

A total of 7,177 genes were identified as dynamically expressed across all three severity groups via EBSeq-HMM analysis (**Table 2**; **Supplementary Table S4**). The Down-Down expressional direction was consistently the most highly expressed path, resulting in 2,232, 1,939, and 1,862 for Healthy, Treated 1, and Treated 2+ cattle, respectively. Likewise, the Down-Down expressional direction demonstrated the highest level of overlap between the three severity groups (*n* = 804; **Figure 2**). Next, the Down-Up expressional direction was the second-most abundant path, resulting in 184, 165, and 379 genes found in the Healthy, Treated 1, and Treated 2+ cattle, respectively. The Down-Up path demonstrated 13 genes to be uniquely shared between all three severity groups: *ALOX5*, *ALOX15*, *ENHO*, *FRRS1*, *HPGD*, *KCTD15*, *LOC100295883* (*CYP4F3*), *LOC100297044* (*CCL14*), *LOC100337044* (*ADGRE3*), *LOC112446413* (*TRGV3*), *MAP6D1*, *SLC7A11*, and *SMPD3* (**Supplementary Table S4**; **Figure 2**). Analysis of the “Up-Down” expressional direction resulted in 163, 112, and 132 genes identified in Healthy, Treated 1, and Treated 2+ cattle, respectively, with no overlapping between all three severity groups. The Up-Up expressional direction resulted in the fewest identified genes, with one, 0, and eight found in the Healthy, Treated 1, and Treated 2+ groups, respectively; no overlap was identified between the Healthy and Treated 2+ group for Up-Up expression.

**Table 2:** Total number of dynamically expressed genes by directionality in each severity group via EBSeq-HMM.

Severity group	Directionality	DEGs
Healthy	Up-Down	163
	Up-Up	1
	Down-Down	2,232
	Down-Up	184
Treated 1	Up-Down	112
	Up-Up	0
	Down-Down	1,939
	Down-Up	165
Treated 2+	Up-Down	132
	Up-Up	8
	Down-Down	1,862
	Down-Up	379

### 3.2 Functional enrichment analyses of differentially expressed genes

**Figure 3:**
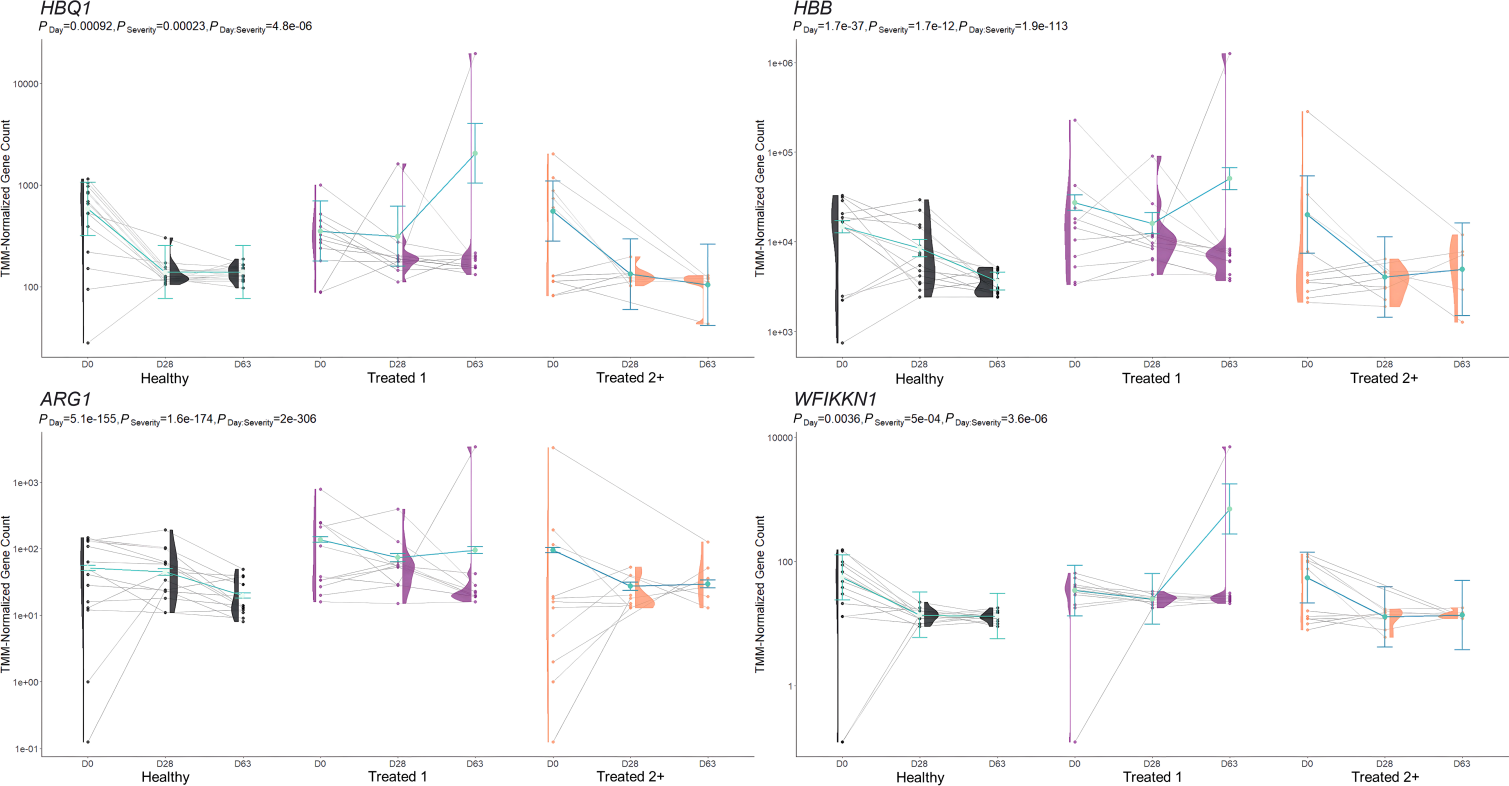
Model plot of key differentially expressed genes identified between Healthy and Treated 1 cattle (*ARG1*, *HBB*, *HBQ1*, and *WFIKKN1*). Gene expression levels of selected genes driving multiple significant enrichments are shown for all three severity groups, indicated by the *x*-axis (black: Healthy, purple: Treated 1, orange: Treated 2+) and further denoted by day (D0, D28, D63). Normalized relative gene expression levels for selected genes are indicated by the *y*-axis. Dots represent the relative gene expression for an individual, spaghetti plot lines indicate the relative trend of gene expression over time for an individual, violin plots represent the distribution of relative gene expression for a severity group at each time point, and overlapping blue lines represent the fitted model utilized by glmmSeq.

In total, 259 GO terms and 134 enriched pathways were identified from differential gene expression analyses via glmmSeq and edgeR (**Supplementary Table S5**). The total number of enriched GO terms and pathways from each comparison is found in **Table 3**. Generally, the most informative time points were at Day 0 and the final collection time point (Day 63); notably, no individual was treated after Day 42 (**Supplementary Table S1**). Regarding Healthy cattle when compared to Treated 1 cattle, no significantly enriched GO terms or pathways were identified for Days 0 and 28. Day 63 resulted in 32 and 26 GO terms and pathways, respectively. These enrichments primarily involved heme scavenging, erythrocyte exchange of O_2_/CO_2_, neutrophil degranulation, autophagy, fatty acid metabolism, metal ion binding, negative regulation of activated T-cell proliferation, and interferon-gamma-mediated signaling, fatty acid metabolism, and neutrophil degranulation. Trend-wise modeling of the genes driving the majority of these significant enrichments is found in **Figure 3**.

**Table 3:** Total number of enriched Gene Ontology (GO) terms and Reactome and KEGG pathways identified through differentially expressed genes discovered in each pairwise comparison via KOBAS-i.

Comparison	GO terms	Pathways
Healthy vs. Treated 1 (Day 0; glmmSeq + edgeR)	0	0
Healthy vs. Treated 2+ (Day 0; glmmSeq + edgeR)	32	38
Treated 1 vs. Treated 2+ (Day 0; glmmSeq + edgeR)	103	21
Healthy vs. Treated 1 (Day 28; glmmSeq + edgeR)	0	0
Healthy vs. Treated 2+ (Day 28; glmmSeq + edgeR)	0	0
Treated 1 vs. Treated 2+ (Day 28; glmmSeq + edgeR)	0	0
Healthy vs. Treated 1 (Day 63; glmmSeq + edgeR)	32	26
Healthy vs. Treated 2+ (Day 63; glmmSeq + edgeR)	1	0
Treated 1 vs. Treated 2+ (Day 63; glmmSeq + edgeR)	53	13
Severity: Time Interaction (glmmSeq + edgeR)	38	36

**Figure 4:**
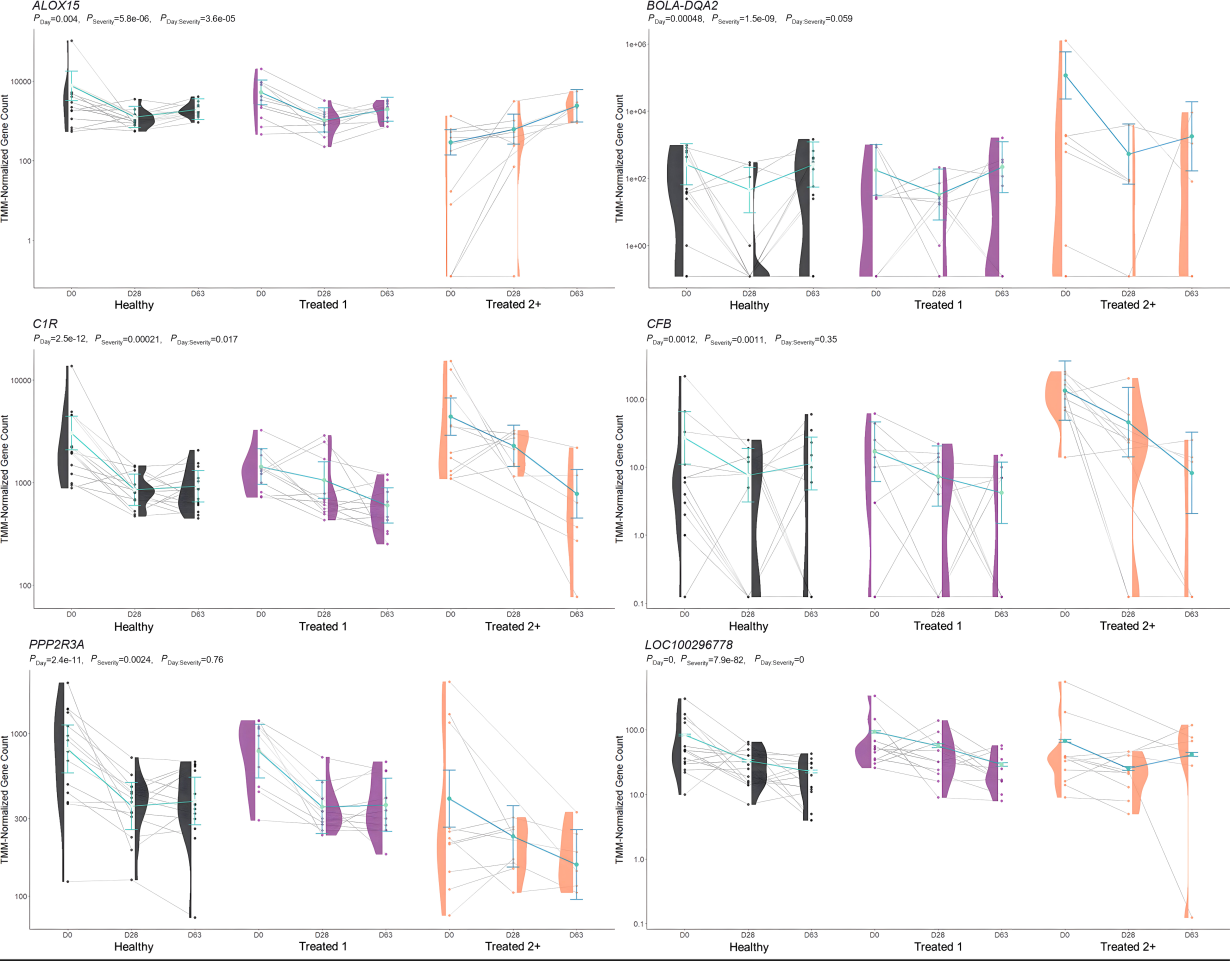
Model plot of key differentially expressed genes identified between healthy and treated 2+ cattle (*ALOX15*, *BOLA-DQA2*, *C1R*, *CFB*, *PPP2R3A*, and *LOC100296778*). Gene expression levels of selected genes driving multiple significant enrichments are shown for all three severity groups, indicated by the *x*-axis (black: healthy, purple: treated 1, orange: treated 2+) and further denoted by day (D0, D28, D63). Normalized relative gene expression levels for selected genes are indicated by the *y*-axis. Dots represent the relative gene expression for an individual, spaghetti plot lines indicate the relative trend of gene expression over time for an individual, violin plots represent the distribution of relative gene expression for a severity group at each time point, and overlapping blue lines represent the fitted model utilized by glmmSeq.

When comparing Healthy and Treated 2+ cattle, significant enrichments for GO terms and pathways were identified at Days 0 and 63. At Day 0, a total of 32 and 38 GO terms and pathways were discovered, respectively; these terms and pathways involved arachidonic acid and linoleic acid metabolism, synthesis of leukotrienes and eoxins, biosynthesis of pro-resolving mediators, antigen processing and presentation, MHC class II protein complex, T-cell differentiation, alternative complement activation, and AMP-activated protein kinase signaling. At Day 63, DEGs were found between Healthy and Treated 2+ cattle enriched for one GO term: natural killer cell-mediated cytotoxicity. Trend-wise modeling of the genes driving multiple of these significant enrichments is found in **Figure 4**.

**Figure 5:**
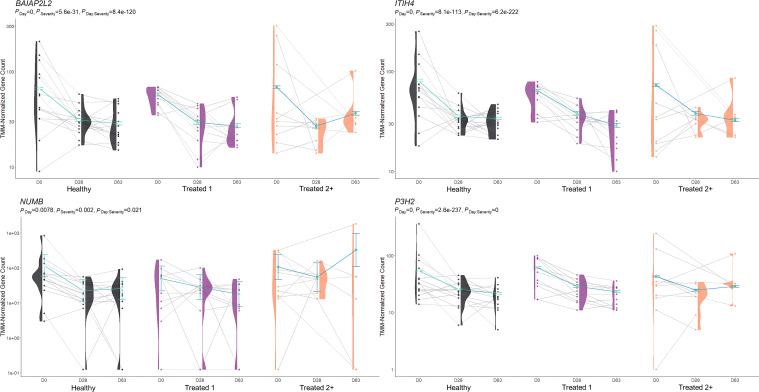
Model plot of key differentially expressed genes identified between treated 1 and treated 2+ cattle (*BAIAP2L2*, *ITIH4*, *NUMB*, and *P3H2*). Gene expression levels of selected genes driving multiple significant enrichments are shown for all three severity groups, indicated by the *x*-axis (black: Healthy, purple: Treated 1, orange: Treated 2+) and further denoted by day (D0, D28, D63). Normalized relative gene expression levels for selected genes are indicated by the *y*-axis. Dots represent the relative gene expression for an individual, spaghetti plot lines indicate the relative trend of gene expression over time for an individual, violin plots represent the distribution of relative gene expression for a severity group at each time point, and overlapping blue lines represent the fitted model utilized by glmmSeq.

The comparison of Treated 1 and Treated 2+ cattle yielded significant enrichments for GO terms and pathways at Days 0 and 63, similar to the aforementioned analyses. At Day 0, 103 and 21 enriched GO terms and pathways were identified, respectively; particularly, these resulted primarily from the nine DEGs also found between Healthy and Treated 2+ cattle at Day 0: *ALOX15*, *BOLA-DQA2*, *CFB*, *LCN8*, *LOC100337053* (*ABCC4*), *LOC112441633* (uncharacterized noncoding RNA), *LOC112445169* (*MORF4L1* pseudogene), *LOC112445170* (*MORF4L1* pseudogene), *LOC509854* (*ABCC4*-like), and *PPP2R3A* (**Figures 1**, **4**). These enrichments involved genes related to bacterial infection, complement, and coagulation cascades (classical and alternative), growth factor binding, smooth muscle cell migration and development, arachidonic acid and linoleic acid metabolism, synthesis of leukotrienes and eoxins, biosynthesis of pro-resolving mediators, antigen processing and presentation, MHC class II protein complex, wound healing, and RNA processing and binding. At Days 63, 53 and 13 enriched GO terms and pathways were discovered between Treated 1 and Treated 2+ cattle, respectively; particularly, several resulted from five DEGs also found between Healthy and Treated 1 cattle at Day 63: *ARG1*, *HBB*, *HBG*, *HBQ1*, and *WFIKKN1* (**Figure 3**). These enrichments primarily involved erythrocyte exchange of O_2_/CO_2_ and heme binding, neutrophil degranulation and innate immunity, autophagy, collagen biosynthesis, cytokine and acute phase responses, and muscle activity and fiber development. Trend-wise modeling of the genes driving these significant enrichments is found in **Figure 5**.

**Figure 6:**
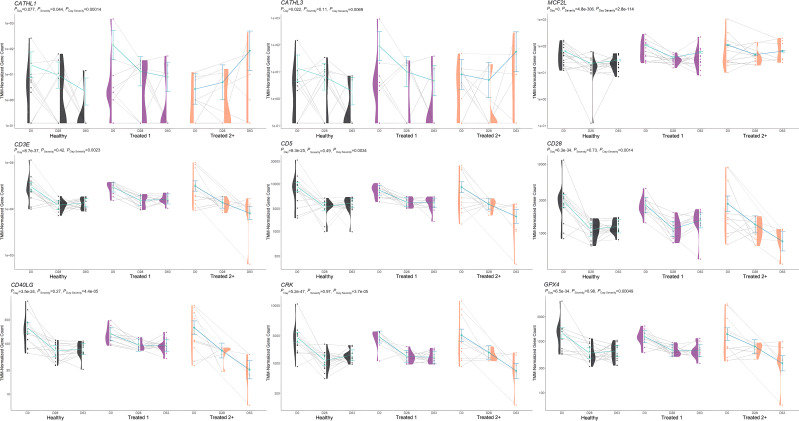
Model plot of key differentially expressed genes identified through the interaction of time and treatment frequency (*CATHL1*, *CATHL3*, *MCF2L*, *CD3E*, *CD5*, *CD28*, *CD40LG*, *CRK*, and *GPX4*). Gene expression levels of selected genes driving multiple significant enrichments are shown for all three severity groups, indicated by the *x*-axis (black: Healthy, purple: Treated 1, orange: Treated 2+) and further denoted by day (D0, D28, D63). Normalized relative gene expression levels for selected genes are indicated by the *y*-axis. Dots represent the relative gene expression for an individual, spaghetti plot lines indicate the relative trend of gene expression over time for an individual, violin plots represent the distribution of relative gene expression for a Severity group at each time point, and overlapping blue lines represent the fitted model utilized by glmmSeq.

Upon evaluation of the DEGs identified through the interaction of Severity and Time, 38 and 36 enriched GO terms and pathways were discovered, respectively. These enrichments are primarily related to RNA processing and metabolism, DNA repair, biosynthesis of pro-resolving mediators, IL-3, IL-5, and CM-CSF signaling, IgA production, T-cell receptor signaling and costimulation, and ubiquitination and tyrosine kinase signal transduction of immune-mediated pathways. Notably, several of these immune-related terms and pathways were enriched by genes previously discovered in the aforementioned analyses, such as *ALOX15*, *ARG1*, *BAIAP2L2*, *HBB*, *HBQ1*, *ITIH4*, *P3H2*, and *WFIKKN1*. Trend-wise modeling of the genes driving these significant immune-related enrichments is found in **Figure 6**.

**Figure 7:**
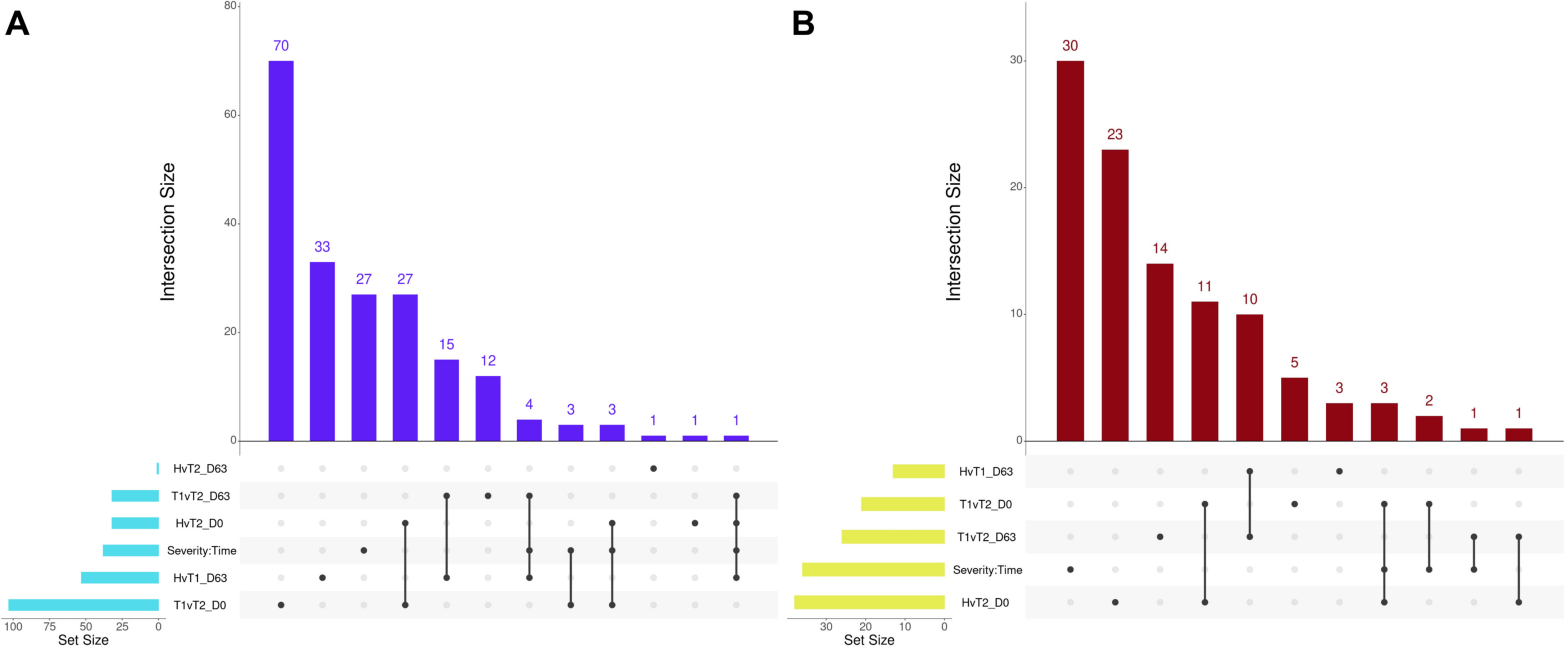
Matrix intersections of enriched Gene Ontology (GO) terms **(A)** and pathways **(B)** identified from differentially expressed genes following pairwise edgeR/glmmSeq analyses. Set size represents the number of enrichments identified between pairwise analyses, and columns represent the number of enrichments (intersection size) corresponding to one or more intersecting sets of analysis (row). Healthy cattle are indicated by “H”, Treated 1 cattle by “T1”, and Treated 2+ cattle by “T2”. Each day of sampling is indicated by “D0”, “D28”, or “D63”.

Several overlapping enrichments were identified between each edgeR and glmmSeq comparison. Regarding Healthy vs. Treated 2+ at Day 0 and Treated 1 vs. Treated 2+ at Day 0, 27 shared GO terms were identified; these terms were primarily related to arachidonic acid/fatty acid metabolism and biosynthesis, MHC class II protein complex, apoptotic cell clearance and antigen presentation, inflammatory regulation and positive regulation of the extracellular signal-regulated kinase (ERK) cascade, and wound healing. Regarding Healthy vs. Treated 1 at D63 and Treated 1 vs. Treated 2+ at D63, 15 shared GO terms were identified; these terms were primarily related to oxygen transport and binding, heme binding, hydrogen peroxide catabolism, muscle fiber development, negative regulation of DNA binding and signaling receptor activity, and endopeptidase inhibitor activity. Regarding Healthy vs. Treated 1 at D63, Treated 1 vs. Treated 2+ at D63, and the interaction of Severity and Time, four shared GO terms were identified; these terms were primarily related to haptoglobin and hemoglobin complex and binding. The number of overlapping GO terms between all edgeR/glmmSeq comparisons is found in **Figure 7A**. With respect to Healthy vs. Treated 2+ at Day 0 and Treated 1 vs. Treated 2+ at Day 0, 11 shared pathways were identified; these pathways were primarily related to bacterial infection, complement and coagulation cascades, and arachidonic acid metabolism. Regarding Healthy vs. Treated 1 at Day 63 and Treated 1 vs. Treated 2+ at Day 63, 10 shared pathways were identified; these pathways were primarily related to heme scavenging, erythrocyte exchange of O_2_/CO_2_, autophagy, and binding/uptake of ligands by scavenger receptors. Lastly, Healthy vs. Treated 2+ at Day 0, Treated 1 vs. Treated 2+ at Day 0, and the interaction of Severity and Time groups resulted in three shared pathways: the synthesis of leukotrienes and eoxins, biosynthesis of specialized pro-resolving mediators, and intestinal immune network for IgA production. The number of overlapping pathways between all edgeR/glmmSeq comparisons is found in **Figure 7B**.

**Figure 8:**
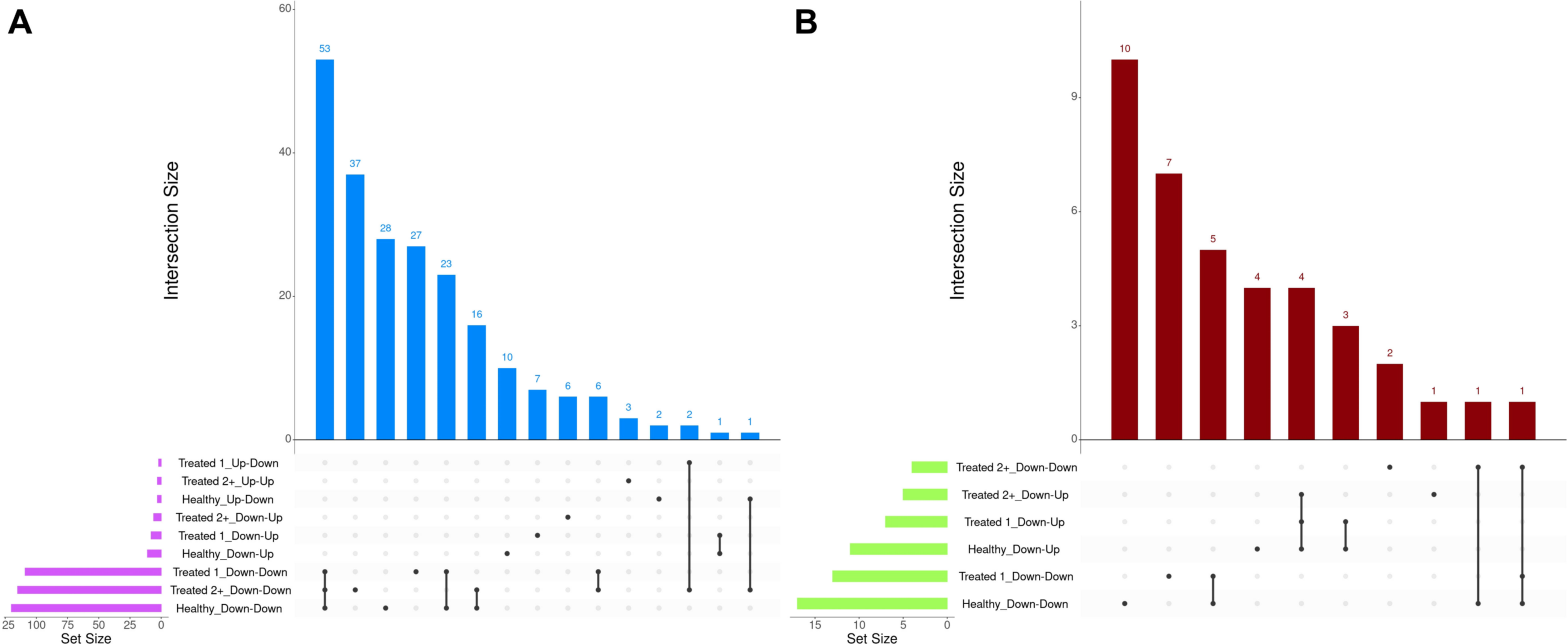
Matrix intersections of enriched Gene Ontology (GO) terms **(A)** and pathways **(B)** identified from dynamically expressed genes following EBSeq-HMM analyses. Set size represents the number of enrichments identified between dynamic expression trends, and columns represent the number of enrichments (intersection size) corresponding to one or more intersecting sets of analysis (row).

A total of 377 GO terms and 57 pathways were identified from dynamically expressed genes discovered through EBSeq-HMM analyses (**Table 4**; **Supplementary Table S6**). Regarding Up-Down gene expression directionality, three GO terms were enriched for Healthy cattle (sex differentiation, development of primary sexual characteristics, and extracellular region), two GO terms were enriched for Treated 1 cattle (regulation of type I interferon production and type I interferon production), and no GO terms were enriched for Treated 2+ cattle. The Up-Up gene expression directionality only demonstrated significant enrichment terms (3) for Treated 2+ cattle: apelin receptor binding, positive regulation of G protein-coupled receptor internalization, and gastrulation. The Down-Down gene expression directionality yielded the greatest number of total functional enrichments across all three disease groups; this is due, in part, to the high number of genes identified in this directionality when compared to the other three (**Table 2**). Moreover, the greatest amount of overlapping enrichments, based on term identification (“term_id”), were found between the three disease groups through the Down-Down gene expression directionality (**Figure 8**). Regarding these overlapping findings, all three treatment groups demonstrated enrichment for GO terms and pathways related to ABC-type transporter activity, ATP hydrolysis and ATP-dependent activity, peptide antigen binding, MHC class II protein complex binding, acid anhydride-acting hydrolase activity, immune and scavenger receptor activity, innate immune response and regulation, cytokine production, defense response to viruses and xenobiotics, cell killing, response to type I interferons, and activation of RAC1 downstream of NMDA receptors.

**Table 4:** Total number of enriched Gene Ontology (GO) terms and Reactome, KEGG, and WikiPathways pathways identified through dynamically expressed genes discovered in each EBSeq-HMM analysis via g:Profiler.

Comparison	GO terms	Pathways
Healthy Up-Down	3	0
Healthy Up-Up	0	0
Healthy Down-Down	120	17
Healthy Down-Up	11	11
Treated 1 Up-Down	2	0
Treated 1 Up-Up	0	0
Treated 1 Down-Down	109	13
Treated 1 Down-Up	8	7
Treated 2+ Up-Down	0	0
Treated 2+ Up-Up	3	0
Treated 2+ Down-Down	115	4
Treated 2+ Down-Up	6	5

Down-Down enrichments unique to Healthy cattle included glycosaminoglycan binding, heparin-binding, sulfur compound binding, collagen fibril organization, adaptive immunity, regulation of multicellular organismal processes, extracellular structure organization, positive regulation of cytokine production, branched-chain amino acid catabolism, translocation of ZAP-70 to immunological synapse, PD-1 signal transduction, and the phosphorylation of CD3/T-cell receptor zeta chains. Specific to Treated 1 cattle, the Down-Down gene expression directionality enrichments included extracellular matrix structural constituent 5′-3′ DNA exonuclease activity, platelet-derived growth factor binding, ion and small molecule binding, Ca-dependent cysteine-type endopeptidase activity, cytoplasmic pattern recognition receptor signaling, myeloid leukocyte-mediated immunity, degradation of the extracellular matrix, integrin cell surface interactions, and collagen biosynthesis and modifying enzymes. Specific to Treated 2+ cattle, the Down-Down gene expression directionality enrichments included lipid transporter activity, positive regulation of leukocyte-mediated cytotoxicity, lymphocyte-mediated immunity, adaptive immune response based on somatic recombination of immune receptors built from immunoglobulin superfamily domains, and calmodulin-dependent protein kinase (CaMK)-mediated phosphorylation of cAMP-response element binding protein (CREB).

Evaluation of enrichments from the Down-Up gene expression directionality resulted in the greatest proportion of overlapping pathways across all three treatment groups. Specifically, all three groups shared Down-Up trends related to the biosynthesis of specialized pro-resolving mediators (resolvins, protectins, and lipoxins), driven by the genes *ALOX5*, *ALOX15*, *HPGD*, and *GPX4* (**Supplementary Table S6**). GO terms unique to the three groups were related to platelet formation and regulation of the mitotic cell cycle in Healthy cattle, carbohydrate phosphatase activity, oxidoreductase activity, Ras protein signal transduction, and biomineral tissue development in Treated 1 cattle, and ferroptosis, negative regulation of wound healing, and regulation of cell morphogenesis in Treated 2+ cattle.

## 4 Discussion

This study was performed as an extension of our previous work, which focused on capturing at-arrival (D0) candidate biomarkers that may predict clinical BRD within the first 28 days after arrival to a growing operation (19, 20, 58, 59). From those studies, we established that genomic mechanisms related to specialized pro-resolving mediator production, type I interferon production and signaling, antiviral defense, alternative complement activation, alpha-beta T-cell complexes, Th2-type immune signaling, and antimicrobial peptide production were associated with later undifferentiated BRD outcomes, both in terms of visually identifiable clinical disease and mortality events. Other studies focusing on molecular approaches to measuring functional host response in association with BRD development and severity have been performed, identifying differentially expressed genes and functional mechanisms related to glucose and heavy metal metabolism, type I interferon production and response, cathelicidins and antimicrobial peptide production, lipoxygenase and serine-type peptidase activity, T-cell differentiation, and immune and inflammatory regulation (18, 22–24, 60–65). Collectively, these findings establish and corroborate several key immune and metabolic host mechanisms as clinically important prognostic indicators of BRD outcomes, which warrants focused research to determine whether the detection and quantification of any of these mechanisms can predict and be used to change BRD outcomes. The findings generated by these studies are highly relevant to current research, as there are no currently recommended biomarkers for predicting or prognosing BRD in commercial cattle production settings (66). Moreover, these studies demonstrate the complexity of undifferentiated BRD and highlight a need for combinational approaches and detection methods (66–69). While our work described here helps corroborate work performed by our group and others regarding potential at-arrival (D0) biomarkers for indicating future BRD development, one understudied element from those studies is evaluating physiological and immunological changes following disease resolution. Ultimately, genomic and diagnostic information related to disease resolution may help improve marketing and management practices, leading to better health for cattle moving into future phases of production, such as feedlot operations.

Importantly, Sun and colleagues in 2020 were one of the first research teams to perform whole blood transcriptome profiling of commercial beef cattle with and without visually observed BRD over multiple time points (18). This study is similar in scope and approach to their work but differs by (1) incorporating previously published data while simultaneously reassessing the at-arrival transcriptomes of cattle not previously sequenced but within the same populations and (2) longitudinally assesses the transcriptomes of cattle after BRD incidence has occurred. Our goals of this study were to further identify and/or corroborate gene expression and enriched mechanisms that indicate BRD development and severity as indicated by treatment frequency and to better understand the impact of BRD development on the host immune system as measured through the blood transcriptome.

While informative, several limitations and opportunities for future research endeavors are apparent from this study. First, the specific pathophysiology of the BRD cases illustrated in this study remains unknown, as none of these cattle underwent antemortem microbial isolation, transthoracic ultrasonography, or metagenomic sequencing of the respiratory tract. As such, the specific nature of the disease remains unclear. However, this is not unusual in commercial operations, as the diagnosis of clinical BRD remains predominantly dependent upon the detection of associated visual signs (65, 70–72). While emerging technologies and combinational approaches for early BRD detection are more routinely being tested and utilized, there is still no biomedical gold standard test for evaluating lung inflammation or pathology, and most novel risk factor associations and promising diagnostic strategies have not been validated prospectively in large-scale populations of cattle (73–77). Likewise, recent research underlines the polymicrobial and multifaceted nature of BRD acquisition, illustrating the lack of causal understanding despite decades of research (69, 78, 79). Collectively, this indicates that BRD is not a “one-size-fits-all” disease process; thus, the disease process and causal nature are most likely different from population to population, and prognostic and diagnostic efforts require a combinational approach. Second, our study utilized two independent populations of cattle. This element of the study serves as both a strength and limitation, as cross-populational studies will likely introduce variation more consistent with commercial cattle operations. However, the second and final sampling time points were different for each year. While studies have demonstrated that *in vivo* host gene expression in cattle is highly influenced by time, physiological development, and their associated microflora, it is still unclear how much variation is seen day-by-day (80–84). Lastly, while this study did not administer antimicrobials to cattle upon arrival (i.e., metaphylaxis), our recent work illustrates how antimicrobials greatly influence host gene expression days and weeks after administration (85). We observed no differential gene expression patterns at Day 28 between the treatment cohorts in this study but were unable to block the antimicrobial treatment effect; the lack of differential expression may be confounded, in part, by the effects of the antimicrobials used in clinical treatment.

### 4.1 At-arrival gene expression is useful in determining the risk of BRD

While relatively anticipated, we identified 40 at-arrival DEGs associated with BRD development within the first 28 days on-feed. First, we were only able to identify four DEGs between Healthy and Treated 1 cattle at arrival: *GSE1* (Gse1 coiled-coil protein; comparatively increased in Healthy), *LOC112446699* (uncharacterized; discontinued the recent update to the *Bos taurus* reference genome assembly), *LOC112446744* (endogenous retrovirus group K member 25 Env polyprotein-like; discontinued the recent update to the *Bos taurus* reference genome assembly), and *LOC112447761* (protein phosphatase 1B-like pseudogene). Ultimately, these genes had no discernible functional enrichment pattern and had not been reported in previous literature focusing on respiratory disease. Similar to our previous work, discernible differences in at-arrival gene expression patterns between Healthy and Treated 1 cattle were somewhat unclear (20, 21). Importantly, BRD diagnosis in this study was made based on visual perception of clinical disease, within semi-objective criteria. However, it is well understood that visual signs associated with BRD are relatively insensitive and nonspecific, limiting the confidence in claiming that both subclinical diseases did not exist in Healthy individuals and that Treated 1 cattle truly possessed lung pathology (11, 72, 85). The exact pathophysiology associated with Treated 1 cattle remains unclear; future research in an effort to better understand lung inflammation and physiological changes in Treated 1 cattle is highly warranted. Nevertheless, previous research suggests that, when observed retrospectively, increased treatment frequencies appear to indicate an increase in confidence for observing a lung pathology event, despite the potential for inconsistent observer agreement (7, 8, 86–88).

When evaluating Treated 2+ cattle, 13 and 23 at-arrival DEGs were identified when compared to Healthy and Treated 1 cattle, respectively. Regarding these findings, nine DEGs were identified between the two analyses and were unique to Day 0: *ALOX15*, *BOLA-DQA2*, *CFB*, *LCN8*, *LOC100337053* (ATP-binding cassette subfamily C member 4), *LOC112441633* (uncharacterized ncRNA), *LOC112445170* (mortality factor 4-like protein 1 pseudogene), *LOC509854* (ATP-binding cassette subfamily C member 4-like), and *PPP2R3A*. Moreover, 27 and 11 enriched GO terms and pathways were shared between the two comparisons, which were related to arachidonic acid/fatty acid metabolism and biosynthesis, MHC class II protein complex, apoptotic cell clearance and antigen presentation, complement activation, inflammatory regulation and positive regulation of the extracellular signal-regulated kinase (ERK) cascade, and wound healing. *ALOX15*, *BOLA*-class genes, *CFB*, *LCN*-genes, and *PPP2R3A* have been identified, with analogous gene expression trends, in previous research related to BRD development in commercial cattle; therefore, the products of these genes in particular warrant further evaluation to test their merit as biomarkers to predict severe BRD, or possibly, to diagnose active airway inflammation (18, 20–22, 89, 90).

*ALOX15* was comparatively decreased in expression at arrival in Treated 2+ cattle when compared to both Healthy and Treated 1 cattle. It encodes for an arachidonic acid lipoxygenase involved in the oxygenation of polyunsaturated fatty acids (PUFAs) and is critical to the generation of PUFA metabolites, namely lipoxins, protectins, resolvins, and maresins, which maintain cellular homeostasis, induce macrophage subtype switching (M1 to M2), enhance the maturation of dendritic cells, and promote cellular clearance of apoptotic debris and self-antigens (91–95). Moreover, there is emerging research that suggests *ALOX15* therapeutic activation and bioregulation are compelling targets for treating sepsis, airway pathology, and systemic inflammation in patients (96–105). While relatively understudied in cattle, previous research has demonstrated the expression and production of *ALOX15* metabolites in polymorphonuclear cells, addressed the balance of leukotriene and lipoxin levels in relation to inflammatory signaling, and demonstrated, *in vivo*, the continuous increased expression over time in young cattle, suggesting its necessary role in immunological development (82, 83, 106–108).

*BOLA-DQA2* was comparatively increased in expression at arrival in Treated 2+ cattle when compared to both Healthy and Treated 1 cattle and encodes for a class II major histocompatibility complex protein that is primarily involved in peptide loading for antigen presentation to CD4+ T cells (109, 110). Previous work has demonstrated that both the copy number, gene polymorphisms, and general heterozygosity of bovine leukocyte antigens (BoLA) are involved in bovine respiratory syncytial virus and bovine leukemia virus clearance, clinical mastitis, and vaccine response (111–118). It may be hypothesized that Treated 2+ cattle arrived with an underlying respiratory infection, hallmarked by the increase in BoLA production on D0, but the causal nature of the increased gene expression seen here remains unknown.

*CFB*, which was increased in expression at arrival in Treated 2+ cattle when compared to both Healthy and Treated 1 cattle, encodes for the single-chain glycoprotein Factor B, the initial molecule in the alternative pathway of complement (119, 120). Upon hydrolysis of C3 and cleavage by factor D, factor B forms a complex that acts upon the surface of xenobiotics and pathogens, opsonizing substances for phagocytosis, further amplifying complement production, and/or leading to the formation of a membrane attack complex, leading to cellular lysis of a target (119–123). Related to respiratory disease, factor B can be synthesized and secreted by alveolar type II pneumocytes, polymorphonuclear cells, and alveolar M1 macrophages and further induced by local or systemic IL-1, IL-6, TNF-α, and/or IFN-γ activity (124–127). Moreover, recent research suggests the overexpression of *CFB* may serve as a prognostic indicator of severe viral lung disease, such as that of SARS-CoV-2 infection or cardiovascular disease (128–132). Similar to BoLA production, increased *CFB* expression in Treated 2+ cattle on D0 may be due to disease at the time of arrival and a non-specific innate immune response to pathogenic infection.

*LCN8* was comparatively increased in expression at arrival in Treated 2+ cattle when compared to both Healthy and Treated 1 cattle, and it encodes for a beta-sheet-rich member of the lipocalin protein family involved in a diverse array of extracellular transport functions (133, 134). Specifically, *LCN8* has been primarily demonstrated as an important transporter protein for reproductive organ function in murine models (135–137). However, recent research suggests *LCN8* may be involved in allergen-induced immunity and autoimmunity (138–141). Furthermore, while low amino acid sequence similarity exists between lipocalin subtypes, they have been shown to form inter-lipocalin protein complexes in mammalian species and may be evolutionarily constructed through gene duplication events; thus, we cannot rule out ancillary immune functionality in association with other lipocalin proteins (142–145).

*PPP2R3A* comparatively decreased in expression at arrival in Treated 2+ cattle when compared to both Healthy and Treated 1 cattle. This gene encodes for a regulatory subunit of phosphoprotein phosphatase 2 and is involved in the negative regulation of cellular development, metabolism, and signal transduction (146, 147). In several models, *PPP2R3A* has been demonstrated as a key regulator in cardiac and pulmonary function and cellular regeneration, and its impairment has been linked to degenerative inflammatory and oncogenic diseases of the pulmonary system (148–152).

### 4.2 BRD severity as measured by treatment frequency influences the bovine transcriptome after apparent disease resolution

When evaluating the host transcriptome after the apparent resolution of clinical BRD (D63), Treated 1 cattle exhibited increased gene expression for oxygen binding and carrier activity, the hemoglobin complex, iron ion binding, transforming growth factor beta (TGF-β) binding and skeletal muscle development, G-protein beta-subunit binding, and neutrophil degranulation activity when compared to Healthy and Treated 2+ cattle. The genes driving the functional enrichments for heme binding and oxygenation (*HBB*, *HBQ1*, and *HBG*) encode for various hemoglobin subunits. Interestingly, low or extreme levels of hemoglobin have been touted as a key predictor of worsening chronic obstructive pulmonary disease (COPD) in humans (153, 154). Likewise, recent research has described how hemoglobin subunits interact with or are expressed by epithelial and immunologically active cells and exhibit antimicrobial functionality (155–157). Furthermore, the genes driving neutrophilic activity and TGF-β binding/skeletal muscle development (*WFIKKN1* and *ARG1*) have been shown to be activated and involved in COPD, pulmonary hypertension, and chronic airway inflammation (158–162). This may signify that underlying airway inflammation and/or infection was actually ongoing in the Treated 1 cattle, even in the absence of outward clinical signs of BRD. If true, this would support the insensitivity of recognizing BRD by visual signs alone and the need to monitor cattle over time using more advanced diagnostic modalities to gain a better understanding of BRD development and resolution. Future research investigating the upper and lower airways of cattle over time, through such modalities as transthoracic ultrasonography, intra-airway fluid cytology, and/or lung biopsy, may better our understanding of BRD pathophysiology.

The comparison of Treated 2+ cattle to Healthy cattle at D63 resulted in only one enriched GO term (natural killer cell-mediated cytotoxicity), despite the identification of 89 DEGs, including immune-related genes such as *CATHL2*, *CCL25*, *CD177*, *LOC112444466* (*SCART1*), *MCF2L*, *PRG3*, and *WC1-8*. Notable, several of these genes have been indicated in previous research utilizing the host transcriptome to indicate or predict BRD, which may indicate prolonged gene expression throughout the feeding period, or perhaps ongoing subclinical disease (18, 20–22, 89, 90). While this single enriched GO term is both valid and informative, the lack of overlapping results from these immune-specific genes potentially conveys the need for further research invested in annotating functional enrichments with *Bos taurus*-specific gene expression datasets. Specifically, cattle are not widely accepted as a model organism in human-focused biomedical research, and functional enrichment terms of agricultural species are often inferred from experiments with model organisms (51–53, 163–167). The two genes driving the enrichment of natural killer cell-mediated cytotoxicity (*LOC509956* [cathepsin G] and *LOC100296778* [killer cell lectin-like receptor subfamily I member 1]), both increased in Treated 2+ cattle when compared to Healthy cattle, are indicated in inflammatory airway diseases such as allergen-induced asthma, COPD, and lung adenocarcinoma, and may serve as prognostic indicators (168–173). Currently, it is unclear if the activation of these genes is due to a prolonged infective state within the airways or if natural killer cell activity serves a protective role postinfection in these severely disease cattle (174). Furthermore, *LCN8*, previously discussed in the analysis of at-arrival transcriptomes, is again upregulated at D63 in Treated 2+ cattle when compared to Healthy cattle. The relationship between this lipocalin, immune system activity, and respiratory disease in cattle is currently unknown, but evidence suggests lipocalins possess immunomodulatory activity and may interact with natural killer cells and innate lymphoid cells in inflammatory responses (175–178).

When investigating the interaction of BRD severity as indicated by treatment frequency and time (Day: Severity in glmmSeq), we identified 286 DEGs, corresponding to 38 and 36 enriched GO terms and pathways, respectively. Here, the primary immunological findings pertain to the biosynthesis of pro-resolving mediators, the IL-3, IL-5, and GM-CSF signaling, IgA production, T-cell receptor signaling and costimulation, and ubiquitination and tyrosine kinase signal transduction of immune-mediated pathways. Further investigation of pairwise gene expression was performed with the genes driving these enrichments to better understand differences over time. First, genes driving the production of specialized pro-resolving mediators, namely *ALOX15*, were downregulated in Treated 2+ cattle at arrival when compared to both Healthy and Treated 1 cattle, but seemingly stabilized to an expressional level that possessed no difference to the Healthy and Treated 1 groups by Day 63. Orr and colleagues in 2015 demonstrated that gene expression related to specialized pro-resolving mediator production and leukotriene synthesis in patients hospitalized for traumatic injuries was significantly different whether patients demonstrated uncomplicated (< 5 days postpresentation) or complicated recoveries (≥ 14 days postpresentation, no recovery by 28 days postpresentation, or death) (179). Corresponding with our study, those investigators demonstrated that these genes were often upregulated within 12 h of traumatic injury presentation in patients that went on to have complicated recoveries, and the ratios of genes involved in lipoxin, resolving, and leukotriene production were the same between the two groups by 28 days posthospitalization (179). While both our work and the research conducted by Orr and colleagues failed to evaluate and capture the interactions of these lipid mediators themselves, these findings may indicate a collective return to homeostasis upon survival of traumatic disease that warrants more focused research (83, 179, 180).

Regarding cathelicidin production, we identified a decrease in *CATHL1* expression at arrival in Treated 2+ cattle when compared to Treated 1 cattle; however, the expression of host antimicrobial peptides *CATHL1* and *CATHL3* was increased in Treated 2+ cattle at D63 when compared to Healthy cattle. Cathelicidins are a class of host-derived peptides involved in broad-spectrum antimicrobial activity and host defense during infectious disease (181, 182). Specifically, Bac1 and Bac7, the peptides encoded by *CATHL1* and *CATHL3*, respectively, are primarily involved in extracellular bacterial killing and may be cytotoxic at high concentrations (181–184). While research has focused on the antimicrobial killing component of these peptides when cattle are faced with pathogenic challenges, the increased expression of these genes after peak incidence of BRD presentation within a population may be indicative of ongoing infectious lung pathology in more severe cases. Lastly, *CD40LG*, *CD28*, *CD5*, and *CRK*, all of which were non-differentially expressed between the three severity groups at arrival but downregulated in Treated 2+ cattle at D63 when compared to Healthy and Treated 1 cattle, encode for critical adaptor proteins and receptors that promote T-cell stimulation and activation (185–189). Coupled with cathelicidin and natural killer cell-mediated cytotoxicity gene expression findings on D63, this suggests that cattle having survived a severe course of BRD (Treated 2+) display a more innate immune-driven response following BRD resolution, perhaps indicating dysregulated immunity over time.

### 4.3 Commercial beef cattle share dynamic patterns of gene expression regardless of BRD development

Through the evaluation of dynamic gene expression trends via Bayesian inferential methodology (EBSeq-HMM), we identified numerous genes and associated enrichments with respect to the four selected directionalities (Up-Down, Up-Up, Down-Down, Down-Up) within each of the three severity groups, independently. Regarding Up-Down directionality, genes found in Healthy cattle did not correspond to apparent immunological function (sex differentiation, development of primary sexual characteristics) and may represent the normal physiology of young, developing male cattle. Up-Down genes identified in Treated 1 cattle corresponded to type I interferon production and regulation. Importantly, we did not identify differential expression of type I interferon-related genes at arrival or any posttreatment time point between the three BRD severity groups. This demonstrates the variability of undifferentiated BRD, as numerous other studies have demonstrated the impact of viral agents and the predictive nature of host antiviral signaling on outcomes associated with BRD (18, 22, 24, 58, 61, 64, 190, 191). Alternatively, differences between the three severity groups regarding transient viral infectivity may have been missed due to the relatively small number of sampling time points. While it is expected that a virulent viral challenge elicits a type I interferon-related response, it is unclear how long this type of immune response persists postexposure. Lastly, regarding Treated 2+ cattle, we failed to identify any genes corresponding with Up-Down directionality in this study. Concerning the Up-Up directionality, the Treated 2+ group was the only group to possess significant genes and enrichments; these enrichments corresponded with apelin receptor binding, the regulation of G protein-coupled receptor internalization, and gastrulation. It is unclear if these enrichments are incidental due to model species annotations, but these genes may be involved in influencing pulmonary inflammatory processes (192–195). In potential future studies, additionally, sampling time points may better indicate these gene expression patterns or those that may have been missed by this study.

The Down-Down directionality netted the greatest number of genes and enrichments across the four expressional directions. While several unique enrichments were identified from GO and pathway terms in each of the three severity groups, the overarching theme and genes driving these enrichments were highly similar and indicative of immunological function and signaling. For example, genes from Healthy cattle concerning the immune system involved heparin binding, collagen fibril organization, the adaptive immune response and positive regulation of cytokine production, PD-1 signal transduction, and the phosphorylation of CD3/T-cell receptor zeta chains; Treated 1 cattle enrichments included platelet-derived growth factor binding, cytoplasmic pattern recognition receptor signaling, myeloid leukocyte mediated immunity, and integrin cell surface interactions; Treated 2+ cattle enrichments included positive regulation of leukocyte mediated cytotoxicity, lymphocyte-mediated immunity, and adaptive immune response based on somatic recombination of immune receptors built from immunoglobulin superfamily domains. Several of the genes driving these “unique” immune-related enrichments (*ARG1*, *BOLA-DQA2*, *CD5L*, *CD36*, *CD177*, *CFB*, *C1R*, *HBB*, *HBQ1*, *IL1R1*, *IL16*, *LOC100296778* (*KLRI1*), *MEIS3*, *MIA*, *MYO18A*, *NUMB*, and *PRG3*) were also identified as DEGs at D0 and D63 by edgeR QLF testing. Moreover, all three treatment groups demonstrated shared immunological enrichments related to ABC-type transporter activity, peptide antigen binding, MHC class II protein complex binding, immune and scavenger receptor activity, innate immune response and regulation, cytokine production, defense response to viruses and xenobiotics, cell killing, response to type I interferons, and activation of RAC1 downstream of NMDA receptors. This seemingly indicates a collective gradual decrease in immunological activity across the three severity groups following physiological pressure originating from commercial sale, transportation to the research unit, and novel penmate comingling.

Lastly, we evaluated the enrichments stemming from Down-Up gene expression directionality across the three severity groups. Here, we identified the greatest number of overlapping enrichments with respect to the total number identified. Genes involved in the synthesis of specialized pro-resolving mediators, specifically *ALOX5*, *ALOX15*, *HPGD*, and *GPX4*, were seen to decrease and then increase over time, regardless of BRD severity. Collectively, the results from evaluating dynamic gene expression trends appear to indicate that the magnitude of gene expression, not the directionality, is most indicative of BRD development and severity.

## Data Availability

The data presented in the study are deposited in the National Center for Biotechnology Information Gene Expression Omnibus (NCBI-GEO), accession number GSE194167. Previously generated data utilized by this study are found in the NCBI-GEO, under accession numbers GSE136176 and GSE161396. R code for statistical analyses of raw gene count data are found at https://github.com/mscott16/2023-Longitudinal-BRD.
